# Revascularization of Chronic Total Occlusion of the Infrarenal Aorta in a Patient with Triple Vessel Disease: Report of a Case Treated by Endovascular Approach

**DOI:** 10.1155/2017/7983748

**Published:** 2017-09-06

**Authors:** Mahek Shah, Soumya Patnaik, Rahul Sinha, Issac Opoku-Asare, Khalid Chaudhry, Sean Janzer

**Affiliations:** ^1^Department of Cardiology, Lehigh Valley Healthcare Network, Allentown, PA 18103, USA; ^2^Department of Medicine, Einstein Medical Center, Philadelphia, PA 19141, USA; ^3^Division of Cardiology, Allegheny General Hospital, Pittsburgh, PA 15212, USA; ^4^Department of Cardiology, Holy Cross Hospital, Silver Springs, MD 20910, USA; ^5^Heart Center of Nevada, Las Vegas, NV 89106, USA; ^6^Division of Cardiology, Einstein Medical Center, Philadelphia, PA 19141, USA

## Abstract

Surgical management of advance aortoiliac occlusive disease is time-tested and a widely practiced strategy, particularly when there is significant coronary artery disease associated with aortoiliac occlusive disease. The technological advances in the field of percutaneous techniques have facilitated the use of nonsurgical endovascular alternatives for peripheral arterial disease in patients with significant comorbidities at high surgical risk, as illustrated in our case report. We report a case of chronic total occlusion of the aorta that was treated percutaneously with endovascular stenting. We also discuss the specific technique used in this procedure.

## 1. Introduction

Aortoiliac occlusive disease (AIOD) is a subset of peripheral vascular disease in which lesions can occur anywhere from the distal aorta to the common femoral arteries. The aorta and the iliac arteries may be involved either independently or in combination. Total occlusion of the infrarenal aorta is rare, with an estimated prevalence of 0.15% according to one autopsy study [1]. In patients presenting with AIOD, total occlusion of the infrarenal aorta has been seen in 3 to 8.5% of the cases [2–4]. AIOD commonly occurs in association with coronary artery disease (CAD). Surgical management of advanced AIOD is time-tested and a widely accepted strategy, particularly when there is significant CAD associated with AIOD. The novel developments in percutaneous techniques in recent years have facilitated the use of nonsurgical endovascular alternatives in many patients with CAD and AIOD. We report a case of acute coronary syndrome with critical limb ischemia (CLI) of the lower limbs due to chronic total occlusion (CTO) of infrarenal aorta and extensive bilateral iliac disease managed by endovascular therapy.

## 2. Case Description

A 54-year-old African American male with a 20-pack-year smoking history, habituated to polysubstance abuse, presented to the emergency department with severe pain, numbness, and progressive weakness in his left lower extremity. He also complained of multiple episodes of exertional substernal chest discomfort. He was being treated for chronic back pain secondary to vertebral disk herniation with radiculopathy. At admission, his vitals were stable with blood pressure 151/68 mmHg, tachycardia 115–120 beats/minute, respiratory rate 16 per minute, and saturation 99% in room air. His right foot was cold and distal pulses were not palpable in both feet. Bedside Doppler arterial signals were present. There was mild sensory loss in the left foot with a chronic nonhealing ulcer at the tip of the great toe. Electrocardiogram revealed ST segment elevation and T-wave inversion in leads II, III, and aVF. Laboratory examination was unremarkable except for elevated troponin-I levels. Transthoracic echocardiogram showed an ejection fraction of 20% with hypokinesis of the anterior, anteroseptal, and apical walls. No thrombus was visualized. Conventional angiography and contrast enhanced computed tomography scan of the chest/abdomen/pelvis revealed complete occlusion of the infrarenal aorta and reconstitution of both iliac arteries via the right and left internal mammary (thoracic) arteries collateralizing bilateral inferior epigastric arteries.

**Figure 1:**
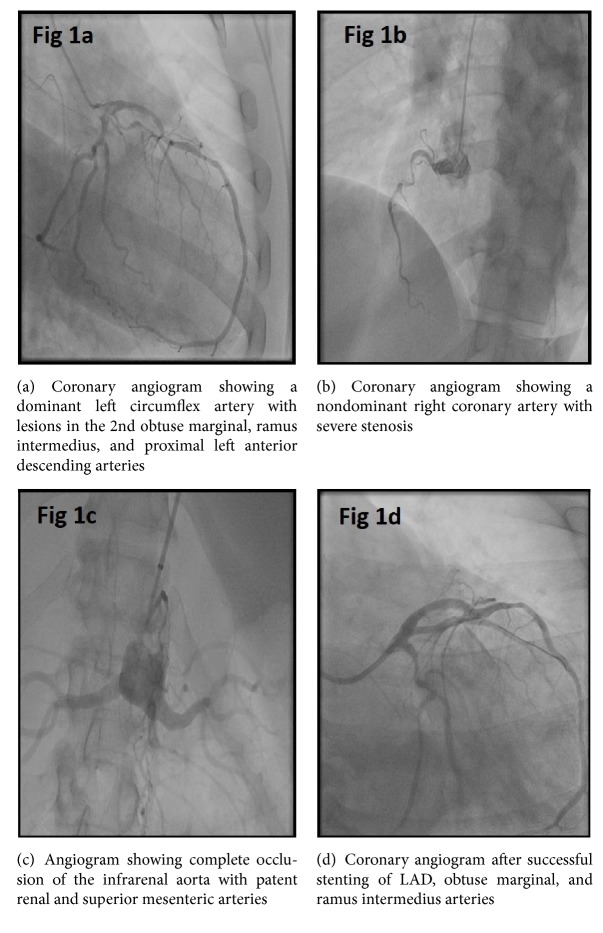


**Figure 2:**
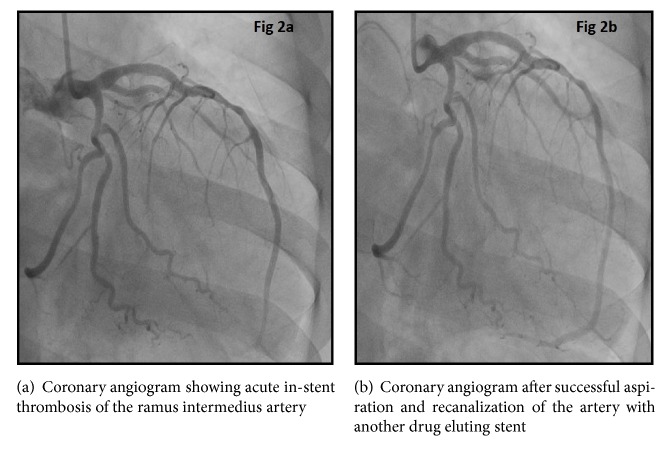


Coronary angiography demonstrated a left dominant coronary system and obstructive lesions were seen in the second obtuse marginal, ramus intermedius, and the proximal left anterior descending (LAD) arteries, as well as the nondominant right coronary (Figures 1(a) and 2(b)). Complete occlusion of the infrarenal aorta with patent bilateral renal arteries and superior mesenteric artery was noted (Figure 1(c)). Successful stenting of LAD, obtuse marginal, and ramus intermedius arteries was performed (Figure 1(d)). 72 hours later, the patient complained of acute chest pain; a repeat coronary angiogram showed acute stent thrombosis in ramus intermedius requiring repeat angioplasty and stent placement (Figures 2(a) and 2(b)).

**Figure 3:**
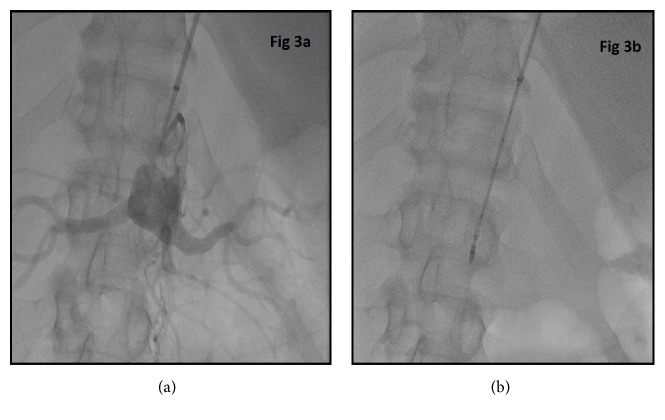
Angiogram demonstrates complete infrarenal aortic occlusion and a Wildcat CTO device is placed within the occlusion. A complex catheterization was performed using the Wildcat looped wire technique.

**Figure 4:**
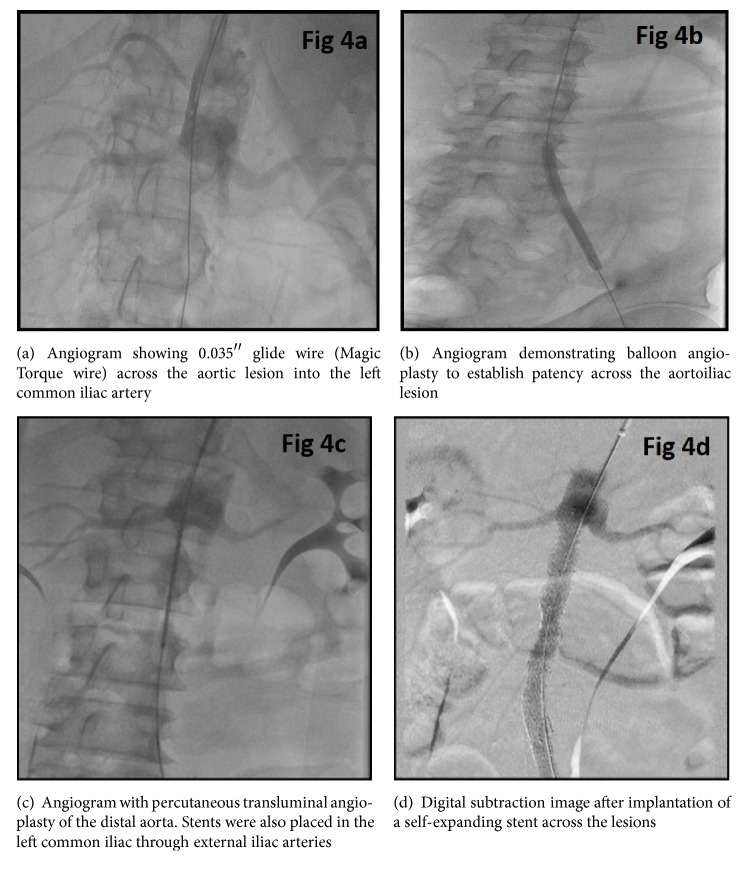


**Figure 5:**
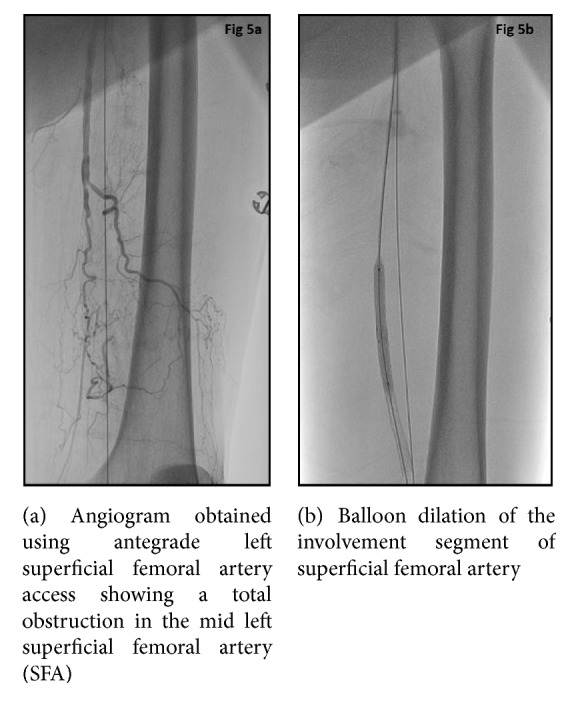


**Figure 6:**
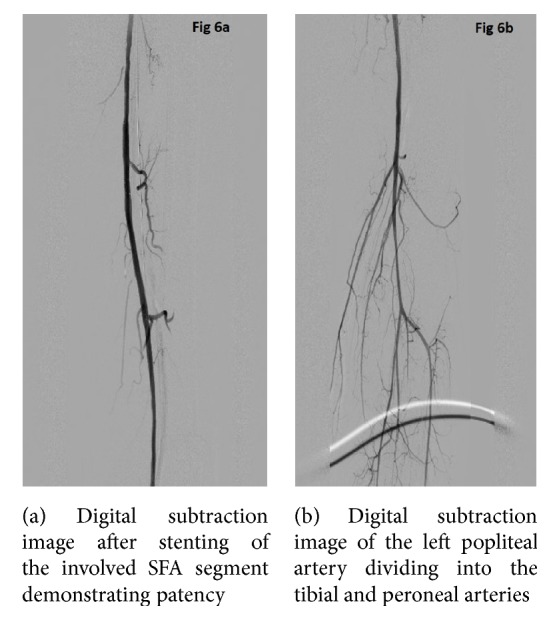


The hospital course was further complicated by recurrent fevers and the patient was found to have worsening rhabdomyolysis within the lower extremities with rising creatinine kinase levels. Patient continued to have fevers despite antibiotics and developed worsening left lower extremity swelling and disappearance of Doppler signals of the left foot. Due to high surgical risk, angiography and a complex percutaneous recanalization procedure were performed. A right common femoral access was attempted but was unsuccessful due to total occlusion in the distal right external iliac artery. After obtaining right brachial access, a 90 cm carotid sheath was placed over a Magic Torque wire and angiogram of the aorta was performed which redemonstrated the CTO of the aorta. As conventional techniques with generic wires and catheters such as 035 glide and Quick-Cross were unsuccessful in crossing the aortic lesion, as a last resort, a Wildcat catheter was selected. After placing a bend on the catheter, the catheter was directed away from the renal arteries and in the direction of the aorta to reduce the risk of perforation. After carefully drilling through the occlusion with the juicebox, the CTO of the distal aorta and left iliac arteries were crossed successfully with a Magic Torque wire. Using a loop wire technique, a Quick-Cross was placed distally and looped down the aorta enabling access to superficial femoral artery with use of a guidewire. Angiography was performed through the Quick-Cross to ensure that it was located in the true lumen of superficial femoral artery. The aortic lumen through the occlusion was then stabilized using self-expanding stents (Figures 3 and 4). The left common iliac, external iliac, and superficial femoral arteries were serially dilated with appropriate sized balloons with good establishment of blood flow to the left lower extremity (Figures 5 and 6).

After the procedure, he remained febrile despite negative cultures and was maintained on empiric broad spectrum antibiotic therapy. However, the infection in the left foot worsened and progressed into wet gangrene, necessitating a left sided above knee amputation. The patient was eventually discharged to a rehabilitation facility after a prolonged in-hospital stay.

## 3. Discussion

Transatlantic Intersociety Consensus (TASC) guidelines, initially published in 2000 and revised in 2007, classify aortic and iliac lesions by lesion morphology (Table 1). TASC II recommends endovascular treatment for TASC A and B lesions and surgical therapy for TASC C and D lesions [5, 6]. Significant CAD that is associated with limb-threatening AIOD have been conventionally treated with coronary bypass surgery followed by appropriate peripheral vascular surgery [7, 8]. The surgery, often comprising anatomic or extra-anatomic bypass grafting or endarterectomy, has been the standard of care often with excellent results. Patency of aortic bifurcation grafts was 90% and 75% at 5 and 10 years in a meta-analysis done in 1997 [9]. Improvements in endovascular therapies for AIOD introduced in recent years offer the advantages of less morbidity, faster recovery, and shorter hospital stay making them competitive alternative to surgery [10–12].

Collateral blood supply via bilateral inferior epigastric arteries and internal thoracic arteries to the lower extremities in our patient meant that using the internal mammary (thoracic) arteries for coronary revascularization would compromise the blood supply to the lower extremity. Hence, the patient underwent percutaneous angioplasty of the stenosed coronary arteries. Critical limb ischemia (CLI) and severe left ventricular dysfunction added to the complexity of the case. CLI independently carries a poor prognosis with high rate of need for amputation (30%) and significant mortality (25%) in 1 year [5]. Acute worsening of lower extremity ischemia in our patient was from the low cardiac output state resulting in downstream ischemia in setting of severe chronic aortic and peripheral vascular disease. An endovascular approach was preferred for recanalizing the aorta in the acute setting, despite the technical difficulties of such a lesion, considering the high risk for surgical mortality. The worsening gangrene of the foot necessitated the emergent endovascular intervention of aortic total occlusion. This procedure is known to be challenging even in the nonemergent setting as the angioplasty is associated with a risk of dissection, perforation, acute thrombosis, atheroembolism, and other contrast related complications (anaphylaxis or contrast-induced renal dysfunction) [13].

Niizeki et al. reported a similar case of patient with CAD with Leriche syndrome, who was relatively stable allowing addressing the aortic occlusion as a first step followed by complex PCI of the RCA lesion. Unlike our patient, the aortic lesion in that patient could be crossed with conventional approach using bifemoral and left brachial approach and two self-expanding stents were deployed at the aortoiliac bifurcation [14]. A study by Kim et al. tackled 49 patients with a CTO of the aorta treated via endovascular approach [15]. They reported procedural success in 40 cases with 7 patients requiring reintervention and an overall 80% patency rate at 3 years. None of the patients required major amputation but major complications were reported in 16% of the cases like spinal and cerebral infarctions, distal embolization, side branch jailing, iliac artery rupture, and pseudoaneurysm formation among others.

**Figure 7:**
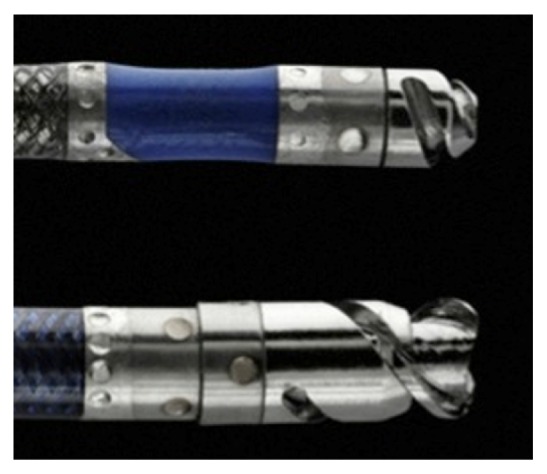
Wildcat catheter tip with a double wedge tip [permission to reproduce the image obtained from Elsevier through Rightslink license 3877131507083 dated 27 May 2016].

Our case also demonstrates the usefulness of a Wildcat catheter to cross the chronic total occlusion (CTO) of the aorta after attempting conventional techniques. The Wildcat catheter was originally designed and tested to cross difficult total occlusion in iliac arteries. It was proven to be safe and effective in crossing total femoroiliac occlusions in the CONNECT Study and was subsequently FDA approved [16]. The device is designed to traverse the CTO through the applications of 2 wedges at the distal tip (Figure 7, adapted from official Avinger website http://avinger.com/products/wildcat). The Frontrunner (Cordis) and Crosser CTO (vibrational angioplasty) are alternative choices though neither of them has been specifically studied for crossing a CTO of the aorta.

When applied to the appropriate anatomical problem, the results of iliac angioplasty/stent placement rival open surgical results but its application in proximal occlusive disease is controversial with lack of long-term follow-up data. Another limitation that may discourage such strategy is the complicated learning curve inherent to the technique. Moreover, no randomized trials have been done, and only single center studies are available in literature.

## 4. Conclusions

Our case illustrates a complex situation of a symptomatic severe CAD with significant LV dysfunction, AIOD, and collaterals from lateral thoracic wall arteries and epigastric arteries to lower limb arteries complicated by distal critical lower limb arterial occlusion that was managed by percutaneous intervention of the coronary lesions and endovascular opening of CTO of aortic occlusion using Wildcat catheter and revascularization of lower limb arterial occlusions.

## Figures and Tables

**Table 1:** TASC classification of the aortic and iliac lesions [permission to reproduce the image obtained from Elsevier through Rightslink license 3877131206925 dated 27 May 2016].

Type A lesions	(i) Single stenosis ≤ 10 cm in length (ii) Single occlusion ≤ 5 cm in length

Type B lesions	(i) Multiple lesions (stenoses or occlusions), each ≤5 cm (ii) Single stenosis or occlusion ≤ 15 cm not involving the infrageniculate popliteal artery (iii) Single or multiple lesions in the absence of continuous tibial vessels to improve inflow for a distal bypass (iv) Heavily calcified occlusion ≤ 5 cm in length (v) Single popliteal stenosis

Type C lesions	(i) Multiple stenoses or occlusions totaling > 15 cm with or without heavy calcification (ii) Recurrent stenoses or occlusions that need treatment after two endovascular interventions

Type D lesions	(i) Chronic total occlusions of CFA or SFA (>20 cm, involving the popliteal artery) (ii) Chronic total occlusion of popliteal artery and proximal trifurcation vessels

CFA: common femoral artery; SFA: superficial femoral artery. Adapted from the TASC II Consensus Document. *Eur J Vasc Endovasc Surg *2007, 33:S1–S70.
